# Effects of Different Drying Methods on Volatile Flavor Compounds in Idesia Polycarpa Maxim Fruit and Oil

**DOI:** 10.3390/molecules30040811

**Published:** 2025-02-10

**Authors:** Hongrui Ping, Yonghui Ge, Wenxuan Liu, Jinxiang Yang, Zhaoxue Zhong, Jinhua Wang

**Affiliations:** 1 College of Food Science And Engineering, Guiyang University, Guiyang 550005, China; 15285355967@163.com (H.P.); skyge@163.com (Y.G.); 13096776237@163.com (W.L.); yjxyjx197@163.com (J.Y.); liuyi4312@gmail.com (Z.Z.); 2 Engineering Technology Research Center for Processing and Comprehensive Utilization of Idesia Polycarpa, National Forestry and Grassland Administration of the People′s Republic of China, Guiyang 550005, China

**Keywords:** *Idesia polycarpa* fruit and oil, drying method, volatile compounds, gas chromatography–ion mobility spectrometry

## Abstract

This study combined gas chromatography–ion mobility spectrometry (GC-IMS) and multivariate statistical analysis to explore the differences in the characteristic aroma of *Idesia polycarpa* Maxim (*I. polycarpa*) fruit and oil under different drying methods: natural drying (ND), hot air drying (HAD), microwave drying (MD), and microwave vacuum drying (MVD). The results revealed that 91 volatile compounds were identified in the fruit, and 82 were found in the oil of *I. polycarpa*. HAD and MD resulted in the most significant loss of volatile aroma in both the fruit and oil. In contrast, MVD demonstrated the best retention of these volatile aromas. Multivariate statistical analysis and odor activity value (OAV) analysis (OAV ≥ 1) were employed to identify 10 volatile aroma compounds considered differentiating factors in the fruit and oil subjected to different drying methods. These compounds, including hexanal, 3-methylbutyric acid, 2-acetylpyridine, guaiacol, valeraldehyde, and butyric acid, significantly contribute to the flavor characteristics of *I. polycarpa* fruit and oil, evoking notes of nuts, caramel, and sourness. The OAVs of these aroma-differentiating compounds in microwave vacuum-dried fruit and oil were higher compared to those from other drying methods. Therefore, when considering the enhancement of volatile flavor compounds, MVD is more effective than the other drying methods in promoting the formation of flavor compounds in *I. polycarpa* fruit and oil.

## 1. Introduction

*Idesia polycarpa* Maxim (*I. polycarpa*), a deciduous woody plant from the Polycarpaceae family, is indigenous to China, South Korea, Japan, and the Russian Far East [1,2]. Relevant research indicates that the oil derived from *I. polycarpa* is rich in higher fatty acids and also contains significant amounts of tocopherols, sterols, squalene, and other natural bioactive substances [3,4]. These constituents are believed to confer upon *I. polycarpa* oil its potential medicinal and edible value. *I. polycarpa* is considered a high-quality woody oil plant and plays a crucial role in enhancing national grain and oil security and promoting sustainable resource utilization. Consistent with China’s commitment to sustainable resource management, the planting area and yield of *I. polycarpa* have been increasing each year. To ensure better storage and transportation, preserve more nutrients and medicinal properties, and improve fruit quality, the primary processing of *I. polycarpa* has become increasingly important.

Drying, as the initial step in processing *I. polycarpa*, is essential for preserving the aroma and quality of the fruit and oil, extending the shelf life, and preventing nutrient loss, color changes, and the formation of oxidation products [5]. Local farmers typically dry *I. polycarpa* naturally by sun drying it after harvest, while businesses primarily use microwave and microwave vacuum drying methods for processing the fruit. The impact of drying methods on the volatile compounds and the quality of *I. polycarpa* can be significant and unpredictable. For instance, Huang et al. (2021) compared the levels of unstable compounds in the leaves of *I. polycarpa* subjected to four different drying methods, finding that sun drying showed the best retention of these compounds in the leaves [6]. Zihui et al. (2024) discovered that infrared vacuum drying and microwave drying significantly affected the quality of the oil, with vacuum microwave-dried oil retaining the highest levels of tocopherol [7]. Zufei et al. (2022) found that vacuum freeze drying produced the highest quality seed oil from *I. polycarpa*, while microwave vacuum drying demonstrated high drying efficiency [8]. Xiaoxiao et al. (2020) reported that the physical and chemical properties of dried *I. polycarpa* seed oil were superior, with minimal loss of trace nutrients [9].

Given the limited studies on how different drying methods affect the volatile aroma compounds in the fruit and oil of *I. polycarpa*, the impact of these methods on the flavor remains unclear. To address this challenge, we employed GC-IMS to systematically compare the volatile components in *I. polycarpa* fruit and oil samples subjected to natural drying (ND), hot air drying (HAD), microwave drying (MD), and microwave vacuum drying (MVD). The objective of this study is to identify the drying conditions that minimize the formation of off-flavors while maximizing the retention of desirable volatile compounds. This study not only aims to enhance the flavor quality and market competitiveness of *I. polycarpa* fruit and oil but also seeks to establish a scientific foundation for standardizing drying processes within the industry.

## 2. Results

### 2.1. Appearance Analysis of I. polycarpa Fruit and Oil Treated with Different Drying Methods

**Figure 1:**
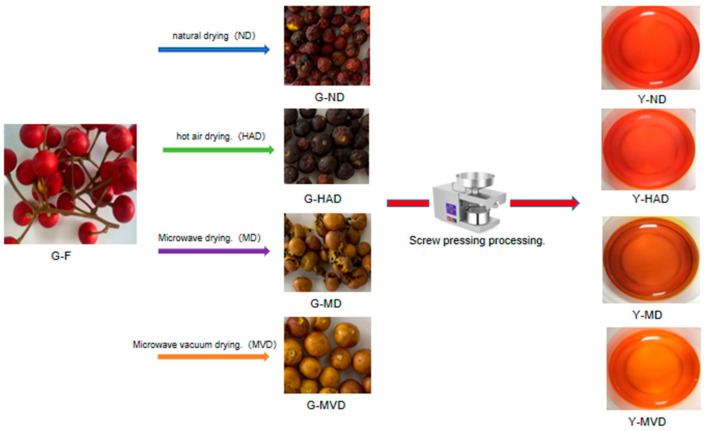
Appearance analysis of *I. polycarpa* fruit and oil treated with different drying methods.

The effects of fruit drying methods on the appearance of *I. polycarpa* fruit and oil are illustrated in Figure 1. The fruit of *I. polycarpa* that was naturally dried (G-ND) exhibited superior color retention, closely resembling that of fresh fruit. In contrast, the fruit of *I. polycarpa* that was hot air-dried (G-HAD), microwave-dried (G-MD), and microwave vacuum-dried (G-MVD) showed more pronounced color changes. This was likely due to the mild drying conditions used, which helped preserve pigment stability and reduce alterations in color caused by the thermal degradation of carotenoids and other pigments [10]. Additionally, the G-ND, G-HAD, and G-MD samples experienced noticeable shrinkage or fragmentation, possibly as a result of water migration and the separation of cytoplasmic walls during slow evaporation [11]. In comparison, the G-MVD samples maintained their shape and color more effectively, likely because the vacuum environment lowers the boiling point of the material, reducing degradation from high temperatures and oxidation [5,12]. In conclusion, the drying methods had a significant impact on the appearance of *I. polycarpa* fruit and oil, with MVD demonstrating superior performance in preserving the shape of the fruit and the color of the oil.

### 2.2. Profile Analysis by GC-IMS

**Figure 2:**
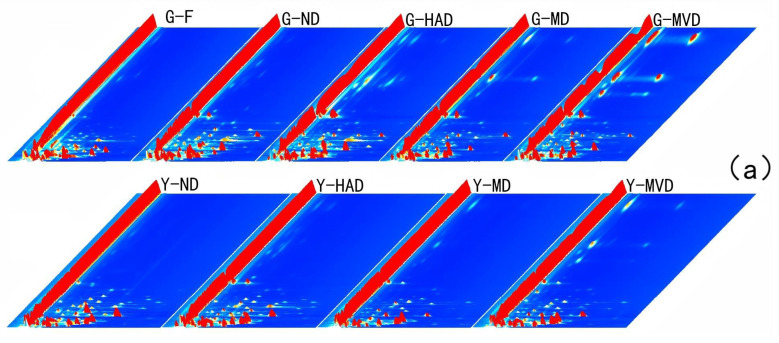

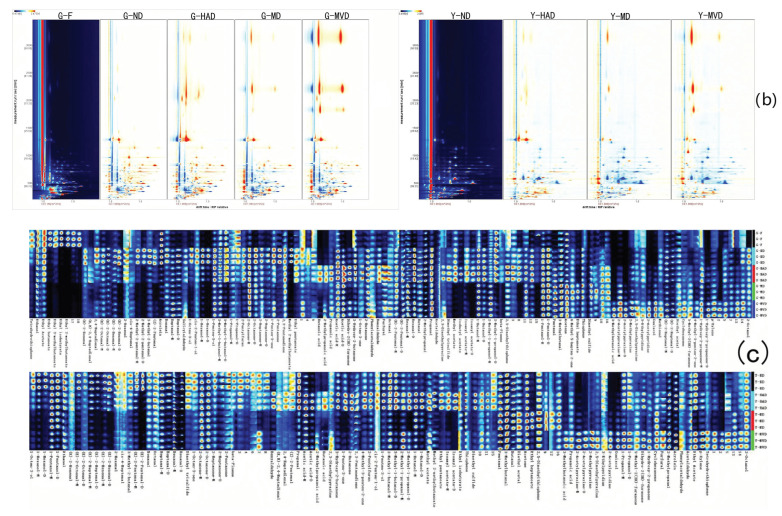
Analysis of *I. polycarpa* fruit and oil subjected to different drying methods using GC-IMS. Three-dimensional topographic maps of *I. polycarpa* fruit and oil obtained after using different drying methods (**a**). Difference comparison plots highlighting variations among samples (**b**). Fingerprints of *I. polycarpa* fruit and oil obtained after using different drying methods, as visualized by Gallery Plot (**c**). Serial number represents undefined substance. Note: “G” denotes fruit of *I. polycarpa*, while “Y” represents oil derived from *I. polycarpa*.

Figure 2a provides a three-dimensional view of the ion migration times, highlighting significant differences among the samples. The two-dimensional representation in Figure 2b further clarifies these differentiations. Since subtle variations in capillary column temperature and carrier gas flow can affect retention time, it is crucial to normalize the reactive ion peak (RIP) to ensure data accuracy. The normalized RIP spectrum displays data points corresponding to volatile compounds, with color intensity indicating concentration, i.e., darker colors signify higher concentrations [13]. The analysis revealed that most signals are concentrated within a retention time range from 100 to 500 s and drift times between 1.0 and 1.5. Although samples processed using the same drying methods exhibited similar peak signal distributions, noticeable differences in peak signal intensities were observed between samples treated with different methods. This suggests that the drying method significantly influences the composition and concentration of volatile compounds in *I. polycarpa* fruit and oil.

### 2.3. Volatile Compounds as Analyzed by GC-IMS

**Figure 3:**
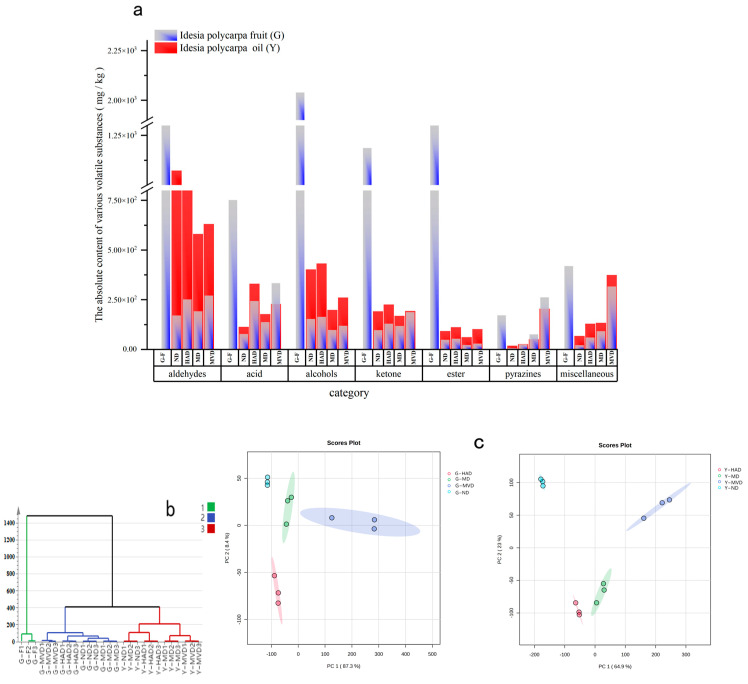
Volatile compound profiling in *I. polycarpa* fruit and oil after using different drying methods. (**a**) Column diagram of volatile compounds. (**b**) Hierarchical cluster analysis (HCA) plot depicts distinct clusters differentiated by color, reflecting similarities in volatile compound profiles. (**c**) Principal component analysis (PCA) score plot presents distribution of samples based on first two principal components, with PC1 explaining 65% and PC2 explaining 24% of the variance.

A comprehensive qualitative and quantitative analysis of the volatile compounds in *I. polycarpa* fruit and oil samples, subjected to four different drying methods, was conducted using GC-IMS technology and the NIST and IMS databases (see Figure 2c). The relative content of volatile compounds across different samples was visually compared using the Gallery Plot plug-in, with color intensity reflecting concentration [14]. The analysis revealed a total of 91 volatile compounds in *I. polycarpa* fruit, including 28 aldehydes, 15 alcohols, 12 esters, 16 ketones, 4 acids, 4 pyrazines, and 12 other categories of compounds. The detailed relative concentrations of these volatile compounds are outlined in Table 1 and Figure 3a. G-MVD demonstrated superior concentrations of aldehydes, acids, ketones, and various other compounds, while G-HAD exhibited elevated levels of aldehydes and alcohols. Importantly, our findings showed that the drying process led to substantial losses of volatile compounds from fresh fruit, particularly in G-HAD and G-MD, where these losses were most significant. This phenomenon is likely due to the combined effects of high temperatures and oxygen exposure during drying, which promote the volatilization of low-boiling-point aroma components and the oxidative degradation of heat-sensitive compounds [15]. Remarkably, we observed increases in 3-methylbutyric acid, 2-ethylpyrazine, 2-ethylpyridine, and guaiacol in G-MVD, possibly resulting from thermally induced Maillard reactions and other thermal processes that enhance the formation of these compounds in low-oxygen environments [16,17]. Our analytical results compellingly indicate that drying methods not only affect the loss of volatile compounds but also contribute to the generation of new flavor compounds. Among all techniques examined, microwave vacuum drying stands out for achieving relatively lower losses of volatile compounds while simultaneously generating desirable new flavor profiles.

A total of 82 volatile flavor compounds were identified in the oil samples (see Table 1 and Figure 3a). Although the oil samples contained nine fewer types of volatile substances compared to the fruit samples, the total volatile content was higher, particularly for aldehydes and alcohols. This increase may result from the disruption of protein structures during the pressing process, which releases volatiles, or from the cleavage of hydroperoxides due to oil oxidation [18]. Notably, pyrazines, acids, and other compounds did not show significant increases in oil samples across the various drying methods. Further analysis indicated that the oil of *I. polycarpa* that was naturally dried (Y-ND) and hot air-dried (Y-HAD) contained elevated levels of aldehydes and alcohols, with similar volatile compound compositions (see Figure 3a). The pressing process of *I. polycarpa* oil enhances the release of more volatile compounds, such as aldehydes and alcohols, which was especially observed in the Y-ND and Y-HAD samples. However, the levels of pyrazine and other thermal reaction products remained elevated in the oil of *I. polycarpa* microwave vacuum-dried (Y-MVD) samples, suggesting that the MVD method may be more conducive to the generation of specific flavor compounds in *I. polycarpa* oil.

### 2.4. Unsupervised Assessment of Volatile Compounds in I. polycarpa Fruit and Oil via Different Drying Methods

Hierarchical cluster analysis (HCA) and principal component analysis (PCA) were conducted to assess the volatile compounds across nine samples of *I. polycarpa* fruit and oil. The aim was to elucidate the effects of different drying methods on the volatilization of these compounds. As illustrated in Figure 3b, HCA categorized the samples into three main clusters: fresh fruit, dried fruit, and fruit oil samples. Notably, the G-MVD cluster was differently separated from the other dried fruit methods, indicating that the MVD method has a unique impact on the volatile compounds. Similarly, in the oil samples, Y-ND and Y-HAD were grouped, while Y-MD and Y-MVD formed a separate cluster. This reinforces the significant influence of drying methods on volatile composition.

PCA further supported the results obtained from HCA. In Figure 3c, the PC1 axis distinguished G-MVD from other dried fruit samples, accounting for 87.3% of the total variance. For the oil samples, as shown in Figure 3c, Y-HAD, Y-ND, Y-MD, and Y-MVD were separated along PC1, which accounted for 64.9% of the variance, while the differences along PC2 explained an additional 23% of the variance. In summary, the results from the PCA and HCA indicate that drying methods significantly alter the volatile profile of the fruit and oil. In particular, using the MVD method for *I. polycarpa* fruit and oil effectively preserves the volatile compounds and promotes the formation of different flavor compounds.

### 2.5. Analysis of Key Differential Volatile Compounds in I. polycarpa Fruit and Oil via Different Drying Methods

**Figure 4:**
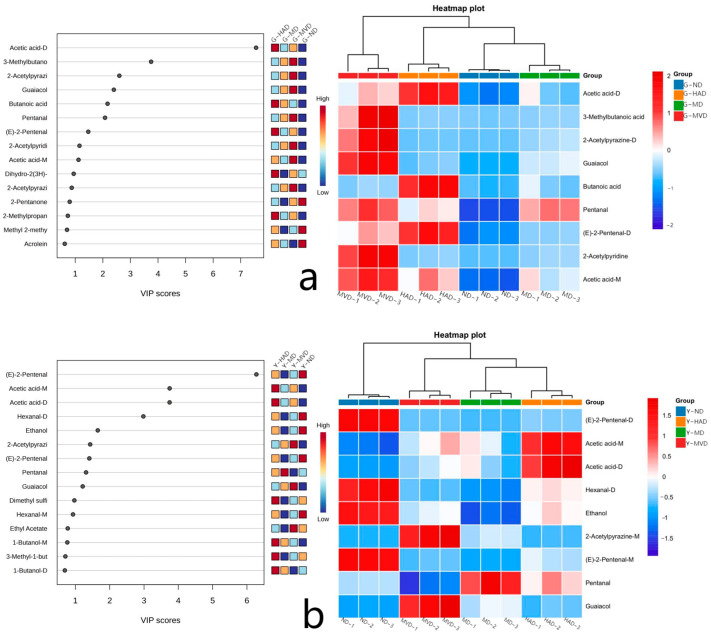
Multivariate statistical analysis: VIP plot and heatmap of critical differential volatile compounds in *I. polycarpa* fruit (**a**) and oil (**b**).

Partial least squares-discriminant analysis (PLS-DA), a supervised statistical method, facilitated the exploration of data correlations and clarified differences in the volatile components of *I. polycarpa* fruit and oil under various drying conditions [19]. In the PLS-DA model, the proximity of R^2^ and Q^2^ to 1 indicated a better model fit. The two established PLS-DA models in this study complied with these requirements (G: R^2^ = 0.866, Q^2^ = 0.803; Y: R^2^ = 0.976, Q^2^ = 0.961). This demonstrated that there was no overfitting in the models, confirming their validity. The variable importance in projection (VIP) score helped identify key volatile compounds that significantly differed among methods. Compounds with VIP > 1 were considered critical, with higher VIP values indicating greater discriminative power. Figure 4a,b identifies nine and ten key volatile compounds in the fruit and oil samples, respectively. In the fruit samples, the G-MVD treatment generally showed the highest concentration of these compounds, except for butanoic acid, which was more concentrated in G-HAD. In the fruit oil samples, Y-ND exhibited higher levels of hexanal-D, ethanol, and 2-pentenal, while acetic acid was more prevalent in Y-HAD. Y-MVD was characterized by higher concentrations of 2-acetylpyrazine-M and guaiacol. These compounds are believed to be the primary contributors to the differences in aroma properties observed between fruit and oil processed using different drying methods.

### 2.6. OAV Analysis of Differential Volatile Compounds

The analysis involving odor activity value (OAV) assesses the contribution of volatile compounds to the overall aroma, taking into account their concentrations and odor thresholds. An OAV exceeding 1 signifies that a substance makes a significant contribution to the sample’s overall aroma, classifying it as a key volatile flavor compound [20,21]. Table 2 presents the OAV for ten key differential volatiles compounds, emphasizing their impact on the aroma perception of *I. polycarpa* fruit and oil when subjected to four distinct drying methods. The findings reveal a notable reduction in the characteristic aroma of fresh fruit post-drying. Notably, hexanal-D, 3-methylbutanoic acid, 2-acetylpyridine, guaiacol, and pentanal—compounds known for their nutty, caramel, creamy, and sour notes—exhibit heightened aroma perception in MVD-treated fruit and oil samples compared to those dried using alternative methods. High concentrations of these compounds are also found in roasted cocoa beans and roasted sesame oil [22,23]. Additionally, butanoic acid, known for its pungent sour taste, shows a more pronounced aroma perception in fruit and fruit oil samples dried using HAD. Butanoic acid is recognized as a key flavor component of butter, primarily resulting from the oxidative decomposition of unsaturated fatty acids. In summary, the drying method substantially influences the volatile composition and flavor characteristics of *I. polycarpa* fruit and oil, with MVD showing particular effectiveness in preserving and enhancing key flavor compounds.

## 3. Materials and Methods

### 3.1. Chemical Reagents and Plant Materials Preparation

2-Octanol with a purity ≥99.0% was supplied by Tianjin KOMIO Chemical Reagent Co., Ltd., Tianjin, China.

Approximately 80 kg of mature *I. polycarpa* fruit was randomly collected from the Mianyang planting area in Sichuan Province. Immediately after harvest, the fruit were transported to the laboratory for moisture content analysis. The moisture content was determined by weighing six randomly selected fresh samples (approximately 50 g each) and baking them in an oven at 105 °C for 24 h until a constant weight was achieved. The resulting moisture content of *I. polycarpa* was found to be approximately 58.26% on a wet basis.

### 3.2. Drying Methods

The *I. polycarpa* fruit samples were randomly grouped and weighed. Four drying methods were applied to process the samples: natural drying (sun drying), hot air drying at 50 °C, microwave drying at 2 kW, and microwave vacuum drying at 2 kW and 0.06 kPa. The selection of drying conditions was based on the practices of growers and processing plants. The drying process continued until the moisture content of *I. polycarpa* decreased to below 7% (on a wet basis), at which point the required drying time was recorded. Each drying condition was tested in three separate replicates (see Table 3).

### 3.3. Extraction Method for I. polycarpa Oil

The oil extracted from *I. polycarpa*, processed via the four drying methods, underwent low-temperature screw pressing at 120 °C. Following pressing, the oil was centrifuged at 5000 r/min for 15 min and then filtered using a suction method.

### 3.4. GC-IMS Analysis Conditions

The volatile compounds in the *I. polycarpa* fruit and oil samples were analyzed using headspace solid-phase microextraction coupled with gas chromatography–ion mobility spectrometry (G.A.S. Gesellschaft für analytische Sensorsysteme mbH, Dortmund, Germany). The detection method involved accurately weighing 1 g of the sample, followed by inoculation at 80 °C for 20 min and subsequently at 85 °C. A 500 μL gas sample was automatically injected into the GC-IMS system via a syringe. Volatile gases were analyzed using a WAX column (15 m × 0.53 mm, 1 μm film thickness, Restek Corporation, Bellefonte, PA, USA), in conjunction with IMS at a temperature of 45 °C. Nitrogen (99.999% purity) was used as the carrier gas at a flow rate of 150 mL·min^−1^. IMS gas chromatography was employed to detect the gas components, with the drift tube maintained at 45 °C for 30 min. The gradient migration program was as follows: 2 mg·min^−1^ for the first 2 min, increasing to 10 mg·min^−1^ within 10 min, escalating to 100 mg·min^−1^ within 20 min, and concluding after 60 min.The qualitative analysis of volatile organic compounds (VOCs) was conducted using the NIST library for GAS, combined with the retention index (RI), IMS database, and drift time (Dt) [25,26]. The RI values were calculated from the retention times of n-alkanes (C7–C30). The relative concentrations of the volatile compounds in *I. polycarpa* fruit and oil were calculated using 2-octanol as the internal standard. The concentration of aroma compounds was determined based on the ratio of the peak area of the aroma compound to the peak area of the internal standard, alongside the concentration ratio of the aroma compound. All samples were analyzed in triplicate.

### 3.5. Odor Activity Value (OAV) Calculation

To evaluate the contribution of aroma compounds to *I. polycarpa* fruit and oil, the odor activity value (OAV) was calculated based on the ratio of each compound’s concentration to its perception threshold. The thresholds for the volatile compounds involved in this study were established based on LJ van Gemert’s Compilation of Taste Thresholds in Water and Other Media (Second Enlarged and Revised Edition), supplemented by comparisons with selected studies. Aroma compounds with an OAV greater than 1 are considered to have a significant contribution to the aroma profile of *I. polycarpa* fruit and oil, whereas those with an OAV less than 1 are regarded as having a minor contribution [19].

### 3.6. Statistical Analysis

SPSS 20 software (IBM Armonk, Armonk, NY, USA) was used to analyze the significance of the data. Origin 2021 software (Origin Lab Corporation, Northampton, MA, USA) was employed to generate histograms, and the MetaboAnalyst platform (https://www.metaboanalyst.ca) was utilized for multivariate statistical analysis (the date accessed on 10 August 2024). Excel was used for data statistics and table creation. A mixed standard of six ketones was detected, and a calibration curve was established to correlate retention time with the retention index. The retention index of each substance was calculated based on the retention time of the target compounds. The GC retention index (NIST 2020) database and the IMS migration time database, incorporated in VOCal software(G.A.S. Gesellschaft für analytische Sensorsysteme mbH, Dortmund, Germany), were used for qualitative analysis and comparison of the targets. Additionally, two-dimensional spectra, differential spectra, and fingerprints of the volatile components were generated using Reporter, Gallery Plot, and other plug-ins within VOCal data processing software. This facilitated the comparison of volatile organic compounds across different samples.

## 4. Conclusions

This study investigated the impact of four drying methods on the aroma profile of *I. polycarpa* fruit and oil. The study demonstrates that the drying method significantly influences the volatile composition of *I. polycarpa* fruit and oil. Specifically, hot air drying (HAD) and microwave drying (MD) result in considerable losses of volatile compounds, while microwave vacuum drying (MVD) minimizes these losses and enhances the production of volatile compounds such as acids, 2-acetylpyrazine, acetypyridine, and guaiacol. Additionally, partial least squares-discriminant analysis (PLS-DA) and OAV analysis were conducted. The key compounds identified, including hexanal, 3-methylbutyric acid, 2-acetylpyridine, guaiacol, and valeraldehyde, significantly contribute to the flavor characteristics of the fruit and its fruit oil, imparting a baking aroma along with nutty, caramel, and sour flavors. Notably, the concentration of these volatile compounds in the samples dried using microwave vacuum methods is more pronounced compared to those dried by other techniques. In summary, the sample of *I. polycarpa* fruit subjected to MVD exhibits a higher volatile content and differential flavor characteristics, rendering this method more suitable for *I. polycarpa* processing compared to other drying technologies. This study not only solidifies the impact of drying methods on the aroma characteristics of *I. polycarpa* fruit and oil but also opens up new avenues for innovative drying technologies. The insights from this study are expected to inspire further research, potentially enhancing flavor profiles in other food products and promoting sustainable food processing practices.

## 5. Discussion

This study’s findings underscore the substantial impact of drying methods on the volatile composition and flavor profile of *I. polycarpa* fruit and oil. Hot air drying (HAD) and microwave drying (MD), due to higher temperatures and oxygen exposure, result in significant losses of volatile compounds, potentially due to accelerated thermal degradation and oxidative reactions. In contrast, microwave vacuum drying (MVD) not only mitigates these losses but also promotes the production of certain volatiles, such as acids, 2-acetylpyridine, and guaiacol, which are crucial for the fruit’s flavor profile. The enhanced production under MVD could be attributed to the method’s ability to create a low-oxygen environment that fosters Maillard reactions and other thermal processes, leading to the formation of these flavor-active compounds. The identified key compounds, including hexanal, 3-methylbutanoic acid, 2-acetylpyridine, guaiacol, and valeraldehyde, significantly contribute to the flavor characteristics of *I. polycarpa*, imparting a complex aroma with notes of nuttiness, caramel, and sourness. The higher concentrations of these compounds in MVD-treated samples suggest that MVD is superior in preserving and enhancing flavor attributes, which could be leveraged to improve market competitiveness. The study’s outcomes have broader implications for food processing, indicating that by adjusting drying conditions, the flavor of food can be optimized. This could lead to the development of food products with tailored flavor profiles, enhancing consumer satisfaction and market potential. Future research should explore the application of MVD across a wider range of food matrices to further enhance flavor and preserve quality, contributing to sustainable food processing practices.

## Figures and Tables

**Table 1:** Identification and quantification of volatile compounds in *I. polycarpa* fruit and oil across different drying methods by GC-IMS.

Number	VOCs	RI	Rt [s]	Dt [RIPrel]	*I. polycarpa* Fruit Samples (mg·kg^−1^)					*I. polycarpa* Oil Samples (mg·kg^−1^)			
					G-F	G-ND	G-HAD	G-MD	G-MVD	Y-ND	Y-HAD	Y-MD	Y-MVD
1	Guaiacol	1909.3	3089.76	1.11957	190.35 ± 31.59 a	6.6 ± 0.36 b	31.76 ± 6.43 b	64.28 ± 5.61 b	230.41 ± 35.53 a	23.18 ± 2.54 c	52.69 ± 6.87 bc	97.13 ± 13.33 b	279.08 ± 36.92 a
2	3-Methylbutanoic acid	1908.2	3082.28	1.48419	47.48 ± 8.44 a	3.34 ± 0.31 b	6.79 ± 0.88 b	10.56 ± 1.3 b	162.63 ± 62.06 b	8.25 ± 1.48 b	8.05 ± 1.26 b	8.14 ± 1.15 b	35.01 ± 6.38 a
3	2-Acetylpyrazine	1745.60	2165.86	1.52123	150.27 ± 19.45 b	4.65 ± 0.74 d	22.07 ± 3.02 cd	73.97 ± 10.86 c	257.46 ± 46.34 a	14.18 ± 0.36 c	21.24 ± 2.07 bc	47.07 ± 5.79 b	196.06 ± 21.78 a
4	Dihydro-2(3 H)-furanone	1710.1	2005.02	1.09032	33.9 ± 4.12 a	6.42 ± 0.5 c	23.46 ± 2.02 b	4.46 ± 1.85 c	19.86 ± 1.13 b	15.41 ± 0.5 b	27.92 ± 1.4 a	9.61 ± 1.3 c	28.57 ± 3.22 a
5	Propanoic acid	1638.6	1717	1.11567	14.89 ± 1.61 a	1.27 ± 0.01 c	4.05 ± 0.41 b	2.3 ± 0.11 bc	4.03 ± 0.34 b	5.02 ± 0.22 c	7.05 ± 0.56 b	6.81 ± 0.53 b	9.74 ± 1.16 a
6	(E)-2-Nonenal	1570.6	1481.55	1.41386	12.76 ± 0.85 a	1.89 ± 0.1 b	1.62 ± 0.08 b	1.16 ± 0.08 b	1.51 ± 0.14 b	5.06 ± 0.39 ab	5.58 ± 0.1 a	4.05 ± 0.41 b	4.3 ± 0.47 b
7	Benzaldehyde	1547.7	1409.78	1.15505	22.86 ± 4.37 a	3.52 ± 0.1 b	7.24 ± 0.8 b	1.61 ± 0.06 b	2.71 ± 0.14 b	3.57 ± 0.13 b	16.61 ± 1.03 a	2.8 ± 0.2 b	3.8 ± 0.24 b
8	(E,E)-2,4-Heptadienal	1517.8	1321.11	1.20476	2.28 ± 0.18 a	0.47 ± 0.01 b	0.43 ± 0.04 b	0.21 ± 0.02 b	0.39 ± 0.04 b	1.67 ± 0.07 b	1.99 ± 0.06 a	1.14 ± 0.06 d	1.36 ± 0.07 c
9	Acetic acid	1503.60	1281.00	1.15848	663.73 ± 134.1 a	70.46 ± 4.6 b	192.11 ± 13.15 b	116.43 ± 14.97 b	153.67 ± 10.98 b	94.55 ± 6.96 c	292.18 ± 28.51 a	154.49 ± 27.6 b	169.95 ± 17.35 b
10	Furfural	1492.8	1251.44	1.09335	5.55 ± 0.51 a	0.42 ± 0.06 d	2.82 ± 0.24 b	1.42 ± 0.22 c	2.41 ± 0.3 b	1.39 ± 0.13 c	2.95 ± 0.48 b	1.75 ± 0.31 c	5.55 ± 0.47 a
11	1-Octen-3-ol	1485.8	1232.44	1.16705	7.16 ± 0.76 a	3.16 ± 0.11 b	1.27 ± 0.04 c	0.46 ± 0.11 c	0.6 ± 0.04 c	10.53 ± 0.3 a	4.94 ± 0.16 b	1.29 ± 0.2 d	2.1 ± 0.17 c
12	2,4-Heptadienal	1485	1230.33	1.2099	8.39 ± 1.47 a	0.86 ± 0.01 b	0.54 ± 0 b	0.23 ± 0.04 b	0.6 ± 0.02 b	1.51 ± 0.16 b	2.26 ± 0.07 a	1 ± 0.11 c	1.05 ± 0.18 c
13	(E)-2-Octenal	1437.30	1109.32	1.82187	34.38 ± 3.59 a	7.63 ± 0.31 b	6.72 ± 0.21 b	5.4 ± 0.2 b	7.03 ± 0.17 b	33.91 ± 0.47 a	17.9 ± 0.64 b	10.72 ± 0.46 c	11.94 ± 0.74 c
14	Nonanal	1400.2	1023.55	1.48376	18.66 ± 3.01 a	1.72 ± 0.06 b	1.16 ± 0.1 b	1.45 ± 0.17 b	1.68 ± 0.08 b	4.78 ± 0.38 a	3.89 ± 0.17 b	3.58 ± 0.05 b	3.32 ± 0.1 b
15	1-Hexanol-M	1369.00	956.55	1.64006	25.68 ± 3.2 a	23.24 ± 0.64 a	11.94 ± 0.86 b	2.73 ± 0.29 c	3.48 ± 0.12 c	42.54 ± 0.45 a	23.77 ± 1.12 b	6.74 ± 0.67 c	8.41 ± 0.51 c
16	Dimethyl trisulfide	1415.1	1057.05	1.30035	2.59 ± 0.31 a	0.26 ± 0.04 c	0.9 ± 0.11 b	0.27 ± 0.01 c	0.28 ± 0.01 c	2.48 ± 0.07 a	2.38 ± 0.13 a	0.91 ± 0.05 b	0.75 ± 0.04 b
17	3-Ethylpyridine	1386.1	992.73	1.10259	6.57 ± 1.24 a	0.4 ± 0.03 b	0.55 ± 0.05 b	0.38 ± 0.01 b	0.74 ± 0.15 b	1.21 ± 0.11 a	1.28 ± 0.11 a	0.97 ± 0.2 a	1.21 ± 0.22 a
18	2,3-Dimethylpyrazine	1350.6	919.03	1.10738	5.63 ± 1.07 a	0.37 ± 0.01 b	0.9 ± 0.11 b	0.4 ± 0.04 b	1.04 ± 0.37 b	1.54 ± 0.04 b	1.86 ± 0.01 a	1.05 ± 0.03 c	1.85 ± 0.12 a
19	(E)-2-Heptenal	1330.70	880.17	1.66717	84.81 ± 15.4 a	7.67 ± 0.44 b	9.67 ± 0.56 b	6.07 ± 0.57 b	14.23 ± 0.92 b	51.04 ± 0.56 a	34.72 ± 1.3 b	11.2 ± 0.43 c	15.52 ± 0.67 c
20	1-Hydroxy-2-propanone	1313.5	848	1.0691	206.31 ± 46.27 a	5.04 ± 0.21 b	12.56 ± 1.54 b	8.54 ± 0.64 b	17.52 ± 1.22 b	6.99 ± 0.03 c	10.68 ± 1.09 b	7.09 ± 0.36 c	15.36 ± 2.28 a
21	3-Hydroxy-2-butanone	1298.7	821.2	1.0691	169.35 ± 41.51 a	5.25 ± 0.21 b	9.49 ± 0.82 b	3.42 ± 1.55 b	5.75 ± 0.44 b	16.58 ± 0.53 b	23.77 ± 1.31 a	6.36 ± 1.63 b	8.43 ± 0.67 c
22	1-Octen-3-one	1308.4	838.62	1.27962	3.33 ± 0.25 a	0.24 ± 0.01 c	0.81 ± 0.17 b	0.54 ± 0.06 bc	0.9 ± 0.09 b	2.88 ± 0.07 a	1.51 ± 0.11 b	1.39 ± 0.1 b	1.08 ± 0.09 c
23	Cyclohexanone	1293.3	811.82	1.15841	5.17 ± 0.98 a	0.23 ± 0.01 c	0.41 ± 0.03 c	0.42 ± 0.03 c	1.16 ± 0.16 b	1.23 ± 0.06 c	2.04 ± 0.08 b	2.57 ± 0.07 a	2.74 ± 0.09 a
24	2,3-Dimethylpyrazine	1295.3	815.1	1.41113	9.5 ± 1.85 a	0.37 ± 0.04 b	0.8 ± 0.07 b	0.51 ± 0.04 b	0.95 ± 0.09 b	2.04 ± 0.03 a	2.07 ± 0.1 a	1.91 ± 0.1 a	1.87 ± 0.09 a
25	(E)-2-Heptenal	1276	783.24	1.0762	11.46 ± 2.95 a	0.35 ± 0.01 b	1.37 ± 0.15 b	0.95 ± 0.2 b	3.15 ± 0.4 b	1.43 ± 0.1 b	1.68 ± 0.08 b	1.34 ± 0.07 b	4.77 ± 0.16 a
26	1-Hydroxy-2-propanone	1263.40	763.05	1.51042	109.95 ± 22.44 a	16.42 ± 0.36 b	20.53 ± 1.21 b	21.55 ± 0.62 b	23.89 ± 0.69 b	69.16 ± 0.36 a	62.1 ± 1.7 b	55.89 ± 2.47 c	35.94 ± 1.03 d
27	3-Hydroxy-2-butanone	1239.3	725.87	1.25256	19.24 ± 4.04 a	3.07 ± 0.04 b	4.45 ± 0.36 b	0.85 ± 0.05 c	1.25 ± 0.02 b	6.22 ± 0.23 b	11.49 ± 0.44 a	4.61 ± 0.14 c	4.76 ± 0.23 c
28	1-Octen-3-one	1230.80	713.13	1.52227	30.98 ± 5.83 a	7.76 ± 0.16 bc	4.97 ± 0.31 c	1.8 ± 0.17 c	3.41 ± 0.21 c	22.12 ± 0.19 a	13.19 ± 0.44 b	3.74 ± 0.2 c	5.72 ± 0.2 d
29	Cyclohexanone	1219.80	697.19	1.48819	269.65 ± 55.57 a	18.9 ± 0.85 b	26.96 ± 1.76 b	6.06 ± 0.76 b	9.36 ± 1.54 b	70.07 ± 0.57 b	93.06 ± 3.03 a	16.91 ± 0.45 c	34.35 ± 1.34 d
30	Octanal	1214.7	689.76	1.18735	3.34 ± 0.62 a	0.34 ± 0.02 b	0.31 ± 0.01 b	0.26 ± 0.02 b	0.32 ± 0.02 b	2.06 ± 0.11 a	1.65 ± 0.03 b	0.95 ± 0.02 b	0.92 ± 0.01 b
31	2-Methylpyrazine	1213.2	687.63	1.09547	11.86 ± 2.03 a	1.23 ± 0.04 b	0.69 ± 0.06 b	0.53 ± 0.09 b	0.83 ± 0.06 b	1.2 ± 0.01 a	1.1 ± 0.08 a	0.46 ± 0.02 c	0.71 ± 0.04 b
32	1-Pentanol	1254.6	749.24	1.14437	1.13 ± 0.29 a	0.09 ± 0.01 b	0.06 ± 0.01 b	0.1 ± 0.01 b	0.14 ± 0.01 b	0.45 ± 0.05 a	0.42 ± 0.02 a	0.4 ± 0.06 a	0.45 ± 0.05 a
33	Heptanal	1195.70	663.20	1.69862	21.61 ± 3.2 a	4.32 ± 0.17 c	5.05 ± 0.36 c	4.11 ± 0.17 c	9.13 ± 0.84 b	26.87 ± 0.42 a	22.74 ± 0.6 b	13 ± 0.73 d	17.08 ± 0.47 c
34	2-Heptanone-D	1191.4	656.83	1.62601	3.82 ± 0.27 a	1.08 ± 0.11 b	0.86 ± 0.04 b	0.61 ± 0.03 b	0.78 ± 0.05 b	4.23 ± 0.02 a	2.47 ± 0.2 b	1.22 ± 0.1 c	1.63 ± 0.1 c
35	1-Penten-3-ol	1175.4	622.91	0.94341	54.43 ± 12.2 a	7.53 ± 0.28 b	9.24 ± 0.62 b	5.27 ± 0.65 b	9.14 ± 0.37 b	21.49 ± 0.1 b	26.41 ± 0.97 a	10.23 ± 0.53 c	11.69 ± 0.4 c
36	1-Butanol-M	1162.6	596.97	1.18075	104.88 ± 23.48 a	5.63 ± 0.31 b	7.89 ± 0.51 b	5.64 ± 0.3 b	7.98 ± 0.3 b	17.87 ± 0.44 d	35.81 ± 1.42 a	25.24 ± 1.52 b	20.21 ± 0.7 c
37	(E)-2-Pentenal	1148.30	569.24	1.36085	331.36 ± 74.22 a	24.1 ± 1.16 b	49.95 ± 3.08 b	35.32 ± 0.27 b	40.32 ± 2.49 b	226.45 ± 1.27 a	32.36 ± 2.83 b	9.61 ± 0.47 d	19.27 ± 0.96 c
38	beta-Pinene	1125.2	527.2	1.21566	14.14 ± 3.38 a	0.65 ± 0.09 b	1.38 ± 0.13 b	0.28 ± 0.04 b	0.25 ± 0.01 b	3.67 ± 0.14 a	3.39 ± 0.04 b	2.53 ± 0.05 d	2.95 ± 0.02 c
39	(Z)-2-Pentenal	1125.7	528.1	1.35806	11.58 ± 3.61 a	3.51 ± 0.08 b	1.19 ± 0.11 c	0.26 ± 0.03 c	0.76 ± 0.09 c	1.42 ± 0.08 a	1.34 ± 0.07 a	0.37 ± 0.03 d	0.57 ± 0.06 c
40	Hexanal-D	1101.8	487.85	1.55771	47.39 ± 10.38 a	23.22 ± 1.21 bc	36.39 ± 2.27 ab	10.55 ± 1.32 c	28.95 ± 3.07 b	159.35 ± 10.88 a	89.73 ± 2.94 b	34.19 ± 3.03 d	55.06 ± 1.64 c
41	1-Propanol-M	1052.5	420.76	1.11095	37.1 ± 8.88 a	2.18 ± 0.04 b	2.75 ± 0.16 b	1.55 ± 0.16 b	2.76 ± 0.09 b	10.17 ± 0.08 b	10 ± 0.37 b	4.76 ± 0.34 c	11.89 ± 0.55 a
42	1-Penten-3-one	1038.6	403.77	1.3064	91.77 ± 19.71 a	10.16 ± 0.33 b	10.49 ± 0.41 b	3.87 ± 0.64 b	12.79 ± 1.22 b	7.5 ± 0.04 b	13.32 ± 0.52 a	1.02 ± 0.07 d	2.37 ± 0.11 c
43	Thiophene	1028.7	392.14	1.04114	27.71 ± 6.29 a	2.22 ± 0.08 b	2.44 ± 0.1 b	3.16 ± 0.21 b	3.12 ± 0.1 b	5.28 ± 0.05 b	5.81 ± 0.11 a	3.98 ± 0.11 b	5.62 ± 0.18 a
44	Pentanal	998.1	358.15	1.41949	57.26 ± 13.46 a	10.01 ± 0.51 c	37.48 ± 2.6 b	47.36 ± 2.08 ab	51.19 ± 2.92 ab	155.19 ± 0.57 c	169.2 ± 5.12 b	192.13 ± 9.01 a	133.2 ± 4.72 d
45	Ethanol	947.1	318.8	1.13887	973.15 ± 222.55 a	44.51 ± 2.05 b	42.62 ± 2.59 b	39.29 ± 3.44 b	39.38 ± 1.26 b	117.86 ± 2.32 a	85.42 ± 2.94 b	50.23 ± 2.36 c	80.18 ± 3.14 b
46	3-Methylbutanal	925.4	303.59	1.39995	219.73 ± 51.49 a	14.97 ± 0.65 b	20.63 ± 1.47 b	23.8 ± 0.59 b	31.04 ± 1.82 b	92.41 ± 1.12 c	104.07 ± 3.75 b	106.85 ± 5.28 b	118.76 ± 4.14 a
47	2-Butanone	913.5	295.54	1.24079	40.92 ± 10.36 a	7.04 ± 0.21 b	10.5 ± 0.9 b	8.46 ± 0.73 b	11.74 ± 0.67 b	6.85 ± 0.04 c	22.85 ± 1.1 a	8.17 ± 0.4 c	11.08 ± 0.45 b
48	Ethyl Acetate	895.7	283.91	1.33712	532.21 ± 112.42 a	21.24 ± 0.94 b	24.98 ± 1.77 b	9.42 ± 0.18 b	12.17 ± 0.45 b	53.89 ± 0.35 a	50.09 ± 1.61 b	6.81 ± 0.14 c	55.23 ± 1.98 a
49	Butanal	888.6	279.44	1.28267	63 ± 14.41 a	5 ± 0.12 b	8.26 ± 0.64 b	7.2 ± 0.56 b	9.64 ± 0.68 b	37.77 ± 0.19 ab	34.72 ± 1.47 b	38.49 ± 1.81 a	23.04 ± 0.88 c
50	Acrolein	865.3	265.13	1.0565	287.83 ± 65.35 a	14.11 ± 0.45 b	5.71 ± 0.63 b	5.18 ± 0.77 b	3.43 ± 0.15 b	25.92 ± 0.2 c	34.29 ± 1.2 b	13.41 ± 0.53 d	36.9 ± 1.12 a
51	Acetone	835.9	248.14	1.11374	411.86 ± 96.52 a	16.64 ± 0.68 b	20.23 ± 1.18 b	47.19 ± 0.64 b	62.49 ± 2.16 b	53.8 ± 0.37 b	50.77 ± 1.75 b	66.59 ± 3.3 a	55.01 ± 1.67 b
52	Propanal	817.9	238.3	1.14027	64.3 ± 16.09 a	11.41 ± 0.31 b	18.09 ± 1.11 b	12.82 ± 0.71 b	20.48 ± 1.15 b	70.91 ± 0.63 a	67.08 ± 2.21 b	26.55 ± 1.28 c	30.45 ± 1.09 c
53	2-Methylpropanal	826.2	242.77	1.27988	57.62 ± 14.42 a	4.27 ± 0.16 b	5.7 ± 0.34 b	2.15 ± 0.33 b	3.56 ± 0.12 b	15.24 ± 0.39 c	29.65 ± 1.02 b	15.93 ± 0.84 c	34.67 ± 1.5 a
54	Methyl acetate	848.5	255.29	1.19332	64.19 ± 14.82 a	5.1 ± 0.25 b	8.72 ± 0.69 b	0.5 ± 0.05 b	0.59 ± 0.03 b	4.75 ± 0.04 c	10.34 ± 0.59 a	3.59 ± 0.25 d	9.09 ± 0.42 b
55	Tetrahydrothiophene	1121	520.05	1.05371	64.12 ± 11.59 a	1.76 ± 0.18 b	0.71 ± 0.04 b	0.67 ± 0.07 b	0.78 ± 0.03 b	0.87 ± 0.05 b	0.91 ± 0.06 b	0.78 ± 0.05 b	1.93 ± 0.06 a
56	2-Methyl-1-propanol-M	1108.9	499.47	1.17517	61.15 ± 15.74 a	3.2 ± 0.38 b	4.35 ± 0.26 b	3.56 ± 0.56 b	5.34 ± 0.18 b	12.85 ± 0.43 b	23.73 ± 0.69 a	7.44 ± 0.41 c	24.37 ± 0.86 a
57	p-Xylene	1144.5	562.09	1.07604	15.15 ± 3.03 a	0.97 ± 0.07 c	1.86 ± 0.11 bc	2.75 ± 0.15 bc	5.37 ± 0.2 b	3.02 ± 0.11 d	8.67 ± 0.48 b	4.42 ± 0.16 c	12.59 ± 0.44 a
58	2-Pentanone	998.1	358.15	1.36085	79.99 ± 18.52 a	13.29 ± 0.65 b	3.52 ± 0.07 b	3.51 ± 0.43 b	4.44 ± 0.06 b	10.88 ± 0.51 a	6.1 ± 0.23 b	6.18 ± 0.33 b	6.25 ± 0.28 b
59	Methyl 2-methylbutanoate	1020.1	382.3	1.52141	20.78 ± 6.91 a	9.95 ± 0.72 b	1.42 ± 0.19 c	0.34 ± 0.02 c	1.3 ± 0.03 c	1.54 ± 0.01 c	3.17 ± 0.08 a	0.9 ± 0.02 d	2.04 ± 0.07 b
60	Ethyl isobutyrate	984.5	346.81	1.56157	313.49 ± 76.6 a	0.6 ± 0.05 b	0.36 ± 0.05 b	0.44 ± 0.01 b	0.41 ± 0.01 b	3.93 ± 0.07 d	6.85 ± 0.01 a	5.56 ± 0.14 c	5.98 ± 0.16 b
61	2,3-Pentanedione	1061.6	432.39	1.20309	46.24 ± 9.17 a	4.76 ± 0.15 b	6.38 ± 0.61 b	5.46 ± 0.49 b	5.86 ± 0.61 b	2.2 ± 0.07 b	3.1 ± 0.15 a	1.96 ± 0.1 b	1.46 ± 0.02 c
62	1-Propanol-D	1053.2	421.66	1.25894	63.67 ± 13.83 a	2.78 ± 0.08 b	2.05 ± 0.26 b	1.46 ± 0.07 b	1.86 ± 0.17 b	2.64 ± 0.07 a	2.89 ± 0.18 a	1.56 ± 0.14 b	2.93 ± 0.13 a
63	Ethyl heptanoate	1339.2	896.61	1.42089	9.39 ± 1.44 a	0.91 ± 0.04 c	0.81 ± 0.06 c	4.09 ± 0.53 b	2.69 ± 0.11 bc	2.44 ± 0.05 b	1.99 ± 0.03 b	12.09 ± 0.85 a	2.99 ± 0.12 b
64	1-Butanol-D	1162.6	596.97	1.38319	12.92 ± 2.27 a	3.27 ± 0.13 bc	6.03 ± 0.6 b	1.64 ± 0.13 c	2.74 ± 0.14 c	5.19 ± 0.14 c	21.83 ± 0.83 a	7.59 ± 0.47 b	4.24 ± 0.07 c
65	2-Heptanone-M	1191	656	1.26173	5.98 ± 1.04 a	1.16 ± 0.06 b	1.33 ± 0.04 b	1.31 ± 0.09 b	1.79 ± 0.04 b	6.66 ± 0.21 a	4.21 ± 0.16 b	2.9 ± 0.2 d	3.55 ± 0.15 c
66	Ethyl pentanoate	1136.2	546.88	1.65962	4.43 ± 1.15 a	0.96 ± 0.08 b	1.31 ± 0.12 b	0.2 ± 0.02 b	0.26 ± 0.02 b	2.92 ± 0.13 b	3.74 ± 0.19 a	0.94 ± 0.08 c	0.93 ± 0.03 c
67	Phenylacetaldehyde	1763.1	2249.87	1.26119	30.01 ± 5.3 a	1.96 ± 0.08 b	3.11 ± 0.1 b	2.45 ± 0.28 b	5.04 ± 0.64 b	3.14 ± 0.08 c	5.12 ± 0.35 b	3.57 ± 0.35 c	6.02 ± 0.29 a
68	2-Acetylpyridine	1663.3	1811.64	1.14644	39.6 ± 4.75 b	1.35 ± 0.05 c	6.76 ± 1.01 c	8.15 ± 1.37 c	68.02 ± 13.33 a	6 ± 0.47 c	6.55 ± 0.74 c	11.47 ± 1.38 b	62.84 ± 8.06 a
69	2-Octanone-D	1292.8	810.91	1.75771	59.04 ± 13.21 a	23.66 ± 0.96 ab	23.25 ± 1.48 ab	25.32 ± 0.75 ab	24.18 ± 0.87 b	53.77 ± 0.77 a	52.37 ± 1.78 a	51.1 ± 2.41 a	52.78 ± 1.65 a
70	Acetaldehyde	771.9	214.81	0.97743	29.68 ± 7.5 a	2.25 ± 0.15 b	4.06 ± 0.17 b	4.02 ± 0.39 b	4.43 ± 0.37 b	21.1 ± 1.19 c	23.67 ± 2.44 a	21.45 ± 2.95 c	22.84 ± 1.59 b
71	Diethyl acetal	911.9	294.47	1.02418	58.16 ± 14.58 a	4.55 ± 0.25 b	5.21 ± 0.24 b	5.88 ± 0.64 b	9.84 ± 0.66 b	17.24 ± 0.56 c	17.42 ± 0.3 c	30.19 ± 1.21 a	23.07 ± 0.68 b
72	Dimethyl sulfide	798.1	227.9	0.95114	38.67 ± 8.75 a	3.03 ± 0.14 c	9.06 ± 0.67 b	11.62 ± 0.26 b	9.49 ± 0.48 b	15.82 ± 0.2 b	42.1 ± 1.21 a	7.91 ± 0.55 d	12.1 ± 0.45 c
73	2-Methyl-1-propanol-D	1112.2	505.01	1.36457	291.49 ± 64.76 a	15.46 ± 1 b	21.09 ± 2 b	1.95 ± 0.4 b	5.13 ± 0.58 b	11.21 ± 0.17 b	32.19 ± 1.09 a	1.39 ± 0.06 c	15.57 ± 0.51 b
74	Hexanal-M	1102.2	488.51	1.27399	102.09 ± 20.53 a	7.01 ± 0.73 b	10 ± 0.7 b	11.89 ± 0.17 b	15.69 ± 0.65 b	107.02 ± 1.89 a	88.19 ± 3.1 b	62.11 ± 1.47 d	77.23 ± 2.22 c
75	5-Methyl-2(3H)-furanone	1432.8	1098.52	1.12363	6.15 ± 0.82 a	0.2 ± 0.04 c	0.87 ± 0.13 c	1.45 ± 0.13 bc	2.32 ± 0.42 b	0.71 ± 0.03 c	1.56 ± 0.13 b	1.74 ± 0.19 b	2.49 ± 0.3 a
76	2,5-Dimethylpyrazine	1333.4	885.49	1.10465	4.01 ± 0.67 a	0.28 ± 0.01 b	0.36 ± 0.02 b	0.25 ± 0.01 b	0.83 ± 0.19 b	1.25 ± 0.04 b	1.1 ± 0.02 b	1.05 ± 0.07 b	2.55 ± 0.52 a
77	2,5-Dimethylthiophene	1194.8	662.01	1.07727	7.7 ± 1.72 a	0.42 ± 0.07 b	0.8 ± 0.08 b	0.61 ± 0.03 b	0.76 ± 0.02 b	3.13 ± 0.31 a	3.1 ± 0.06 a	3.51 ± 0.2 a	3.28 ± 0.13 a
78	4-Methyl-3-penten-2-one	1129.3	534.46	1.11143	10.89 ± 5.04 a	0.32 ± 0.03 b	0.32 ± 0.04 b	0.27 ± 0.04 b	1.51 ± 0.25 b	1.97 ± 0.06 b	3.32 ± 0.08 a	0.89 ± 0.1 c	0.95 ± 0.09 c
79	Butanoic acid	1732.8	2106.54	1.16091	14.49 ± 2.28 b	1.43 ± 0.48 c	28.66 ± 3.83 a	4.92 ± 2.51 c	4.56 ± 0.57 c	2.93 ± 0.11 b	14.61 ± 2.12 a	4.01 ± 1.01 b	4.86 ± 0.27 b
80	2-Methylpropanoic acid	1694.4	1937.86	1.15733	10.77 ± 0.46 ab	1.01 ± 0.07 c	12.24 ± 1.68 a	2.77 ± 0.66 c	8.8 ± 1.66 b	2.69 ± 0.18 b	9.14 ± 1.06 a	4.39 ± 0.74 b	8.91 ± 0.86 a
81	Isoamyl acetate-M	1137.60	549.43	1.74924	29.68 ± 6.13 a	3.98 ± 0.4 c	8.92 ± 1.73 b	0.58 ± 0.07 c	0.94 ± 0.02 c	5.34 ± 0.05 b	17.55 ± 0.59 a	1.36 ± 0.2 c	2.43 ± 0.09 c
82	cis-2-Penten-1-ol	1340	898.14	0.94214	22.86 ± 4.32 a	2.2 ± 0.09 b	2.12 ± 0.15 b	1.27 ± 0.01 b	2 ± 0.16 b	5.93 ± 0.09 a	5.92 ± 0.14 a	4.22 ± 0.34 b	4.48 ± 0.23 b
83	1-Hydroxy-2-propanone	1314.8	850.43	1.23568	7.08 ± 1.15 b	0.69 ± 0.02 c	3.6 ± 0.77 c	1.86 ± 0.27 bc	13.45 ± 2.77 a				
84	Salicylaldehyde	1823.6	2565.27	1.13461	37.03 ± 3.05 a	6.79 ± 0.06 bc	8.03 ± 0.42 bc	3.61 ± 0.22 c	10.91 ± 2.49 b				
85	Methional	1476.8	1208.66	1.09635	9.09 ± 1.58 a	0.83 ± 0.04 b	1.03 ± 0.11 b	0.55 ± 0.06 b	1.45 ± 0.15 b				
86	6-Methyl-5-hepten-2-one	1349.4	916.72	1.18109	4.85 ± 0.55 a	0.62 ± 0.01 b	0.96 ± 0.07 b	1.2 ± 0.07 b	1.19 ± 0.08 b				
87	2-Methyl-2-pentenal-M	1186.7	646.68	1.52366	18.85 ± 4.83 a	3.81 ± 0.48 b	0.87 ± 0.05 b	0.35 ± 0.04 b	0.55 ± 0.01 b				
88	Ethyl 2-methylbutanoate	1069.1	442.06	1.65157	182.85 ± 52.22 a	0.17 ± 0.01 b	0.27 ± 0.01 b	0.26 ± 0.01 b	0.35 ± 0.03 b				
89	Ethyl butanoate	1055	423.99	1.55874	70.87 ± 17.63 a	0.57 ± 0.03 b	0.38 ± 0.03 b	0.12 ± 0 b	0.17 ± 0.01 b				
90	Ethyl 3-methylbutanoate	1083.1	460.72	1.26913	36.22 ± 8.83 a	0.33 ± 0.03 b	0.3 ± 0.01 b	0.11 ± 0.01 b	0.16 ± 0.02 b				
91	Isobutyl acetate	1030.3	394.04	1.61523	4.22 ± 0.65 a	0.19 ± 0.01 b	0.99 ± 0.36 b	0.12 ± 0.01 b	0.15 ± 0.01 b				

Notes: The same letter indicates that there is no significant difference in volatile substances between samples (*p* > 0.05), and different letters indicate that there is a significant difference in volatile substances between samples (*p* < 0.05).

**Table 2:** OAV analysis of critical differential volatile compounds.

Numbering	Compound	Odor Threshold (mg·kg^−1^)	Flavor Characteristics	OAV (mg·kg^−1^)								
				*I. polycarpa* Fruit Samples					*I. polycarpa* Oil Samples			
				G-F	G-ND	G-HAD	G-MD	G-MVD	Y-ND	Y-HAD	Y-MD	Y-MVD
1	Hexanal	0.300	Grass aroma	340.3	23.4	33.3	39.6	52.3	356.7	294.0	207.0	257.4
2	(Z)-2-Pentenal	1.500	Fruity and sweet aroma	7.7	2.3	0.8	0.2	0.5	1.0	0.9	0.2	0.4
3	Butanoic acid	2.500	Acid odor	5.8	0.6	11.5	2.0	1.8	1.2	5.8	1.6	1.9
4	3-Methylbutanoic acid	1.800	Irritating rancid odor	26.4	1.9	3.8	5.9	90.4	4.6	4.5	4.5	19.5
5	Ethanol	50.000	Aroma of wine	19.5	0.9	0.9	0.8	0.8	2.4	1.7	1.0	1.6
6	2-Acetylpyridine	0.500	Baking aroma	79.2	2.7	13.5	16.3	136.0	12.0	13.1	22.9	125.7
7	Guaiacol	3.000	Smoked incense and burnt [24] flavor	63.5	2.2	10.6	21.4	76.8	7.7	17.6	32.4	93.0
8	Acetic acid	100.00	Irritating rancid odor	5.7	0.4	0.6	0.5	0.7	0.8	1.8	1.2	1.3
9	Pentanal	17.5	-	13.6	2.4	8.9	11.3	12.2	37.0	40.3	45.8	31.7

Notes: The thresholds for volatile compounds in water were derived from the literature, specifically LJ van Gemert’s “Compilation of Taste Thresholds in Water and Other Media” (Second Edition, Enlarged and Revised). Considering that the monomer and dimer represent two distinct ionic states of the same substance and share identical sensory thresholds and aromatic profiles, the calculation of the OAV for key volatile substances includes the combined concentrations of both the monomer and dimer.

**Table 3:** Sample codes for *I. polycarpa* fruit and oil treated with different drying methods.

Sample Code	Drying Type	Types of *I. polycarpa* Samples	Condition
G-F	Not dried	fresh fruit	-
G-ND	Natural drying (sun drying)	fruit	The *I. polycarpa* fruit was naturally dried after harvest.
Y-ND		oil	Spiral press.
G-HAD	Hot air drying	fruit	50 °C.
Y-HAD		oil	Spiral press.
G-MD	Microwave drying	fruit	2 kW.
Y-ND		oil	Spiral press.
G-VMD	Microwave vacuum drying	fruit	2 kW and 0.06 kPa.
Y-VMD		oil	Spiral press.

## Data Availability

All relevant data are included in the article.
